# Diversity and Bionomics of Sandflies (Diptera: Psychodidae) of an Endemic Focus of Cutaneous Leishmaniasis in Zagora Province, Southeast of Morocco

**DOI:** 10.1155/2021/8812691

**Published:** 2021-01-22

**Authors:** Zalalham Al-Koleeby, Ahmed El Aboudi, Souhail Aboulfadl, Chafika Faraj

**Affiliations:** ^1^Laboratory of Medical Entomology, National Institute of Hygiene, Rabat, Morocco; ^2^Plant and Microbial Biotechnology, Biodiversity and the Environment, Faculty of Science, Agdal University, Rabat, Morocco

## Abstract

The diversity and seasonality for sandflies were studied in 2019 at a focus of zoonotic cutaneous leishmaniasis in Zagora province, southern Morocco. Standardized sampling with CDC light traps was used. A total of 4504 sandflies (4024 *Phlebotomus* and 480 *Sergentomyia*) was collected during the study period. Seven species belonging to genus *Phlebotomus* and six species of genus *Sergentomyia* were identified. The most abundant species were *Ph. papatasi* (33.6%) and *Ph. longicuspis* (25.7%), highlighting the risk for local disease transmission foci. The seasonal activity of sandflies extended from April to November, showing two peaks, one in June-July and one, less important, in late-September-October. Abundance was highest during the months May, June, and July and lowest in August, September, and October. Results of this study provide important baseline data for planning control interventions.

## 1. Introduction

Leishmaniasis is endemic in Morocco with three distinct parasitic species, *Leishmania major*, *L. tropica*, and *L. infantum*, and 2 disease forms, cutaneous and visceral leishmaniasis. Zoonotic cutaneous leishmaniasis (ZCL) caused by *L. major* and anthroponotic cutaneous leishmaniasis (ACL) caused by *L. tropica* are the most widespread manifestations of the disease [1]. Major epidemics have occurred recently, according to the National Leishmaniasis Control Program (NLCP); 53193 CL cases were reported between 2008 and 2017 nationwide. ZCL and ACL account, respectively, for 56% and 44% of recorded cases. This figure does not reflect the real epidemiological situation since the proportion of the cases detected relative to the estimated cases does not exceed 35% [2]. In 2018, the NLCP reported, respectively, 8901 and 2909 cases caused by both *L. major* and *L. tropica* [1].

Cutaneous leishmaniasis caused by *L. major* is endemic in the south and the east of the country where *Ph. papatasi* is the proven vector [3]. The gerbil *Meriones shawi* is the reservoir host in populated areas [4], but it is suggested that there is a “sylvatic” reservoir system that “feeds” this urban system, with the *Psammomys obesus* reservoir [5].

More than 80% of the reported ZCL cases are clustered in the region of Deraa Tafilalt in the southeast of the country made up of five provinces (Errachidia, Tinghir, Midelt, Zagora, and Ouarzazate). Among the 8901 cases identified in 2018, 5675 cases (64%) were diagnosed in the province of Zagora, located in the south of the region. Most of cases are concentrated in rural areas where public health human resources and infrastructure are limited [6, 7]. Surveillance data indicate that the nationwide number of ZCL cases has increased during the last years; such increases can be explained in part by improved diagnosis and case notification but are also a result of inadequate approach to disease control. Indeed, the impact of control interventions is very limited in time since efforts and resources are only mobilized in the event of an epidemic situation, which does not assure the sustainability of the actions and results.

Control measures against ZCL in Morocco rely mainly on case management and rodent reservoir control. The standard of care for prevention of disease transmission is environmental management including promotion of improved solid waste disposal practices [8]. Consideration of vector control is important in this case for successful control of leishmaniasis in Morocco. For this reason, knowledge of phlebotomine ecology is necessary. However, data on leishmaniasis vector dynamics in Moroccan ZCL foci are absent, and most research encompasses only the vectors' seasonal abundance in ACL foci [9–17].

The data presented in this paper provide information on the ecology of the most common phlebotomine sand flies in the municipality of Tinzouline, the most important ZCL focus in Zagora province, southeastern Morocco. Results of this study may help to establish effective and appropriate vector control measures by providing information on abundance and seasonal trend of sandfly species in this active focus.

## 2. Materials and Methods

### 2.1. Study Area

**Figure 1:**
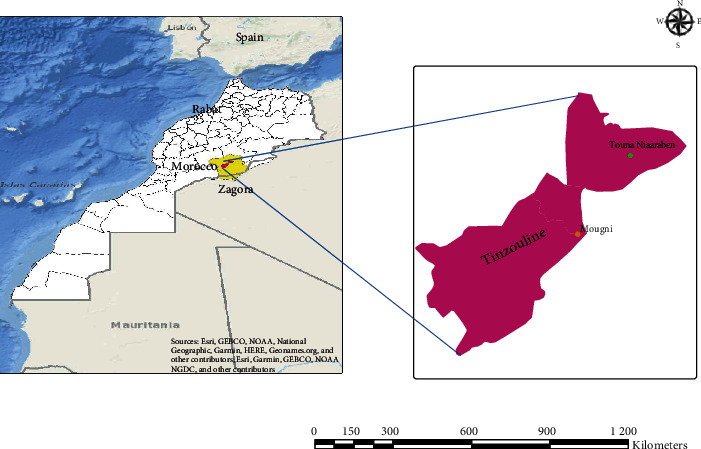
Map showing the location of the study sites.

The study was conducted in Tinzouline, a rural municipality in the province of Zagora (30°30′13.8^″^N 6°06′09.4^″^W) in southeastern Morocco. Investigations were conducted from April to November 2019, in two villages: Touna Niaaraben situated at an altitude of 910 m at around 30°37′28.2^″^N 5°49′56.1^″^W in the northeast and Ksar Mougni situated at an altitude of 775 m at 30°27′3^″^N, 5°58′26^″^W in the east (Figure 1). Tinzouline climate is typically Saharan, hot in summer with an annual mean maximum temperature of 35°C and cold in winter (temperature ranging from −1°C to −7°C). The rainy season occurs from September to May, with 30-40 rainy days annually and an average annual rainfall of 26 mm at Touna Niaaraben and 37 mm at Ksar Mougni.

### 2.2. Sandfly Collection and Identification

We used CDC light traps to collect sandflies. Two traps were set, in fixed locations, indoor and outdoor in five randomly selected habitations, within the two study villages. Sandflies were collected bimonthly during the sandfly activity season from April to November 2019. During each collection session, trapping was performed over two consecutive nights where traps were operated overnight. The insect catch was checked each morning, and sandflies were sorted and kept in 96% ethanol. Caught sandflies were counted, sexed, and identified using morphological keys [18] relying on specific morphological features of the pharynx and genitalia (spermathecae in females, external genitalia in males).

### 2.3. Data Analysis

To characterize the sandfly populations, we calculated four parameters.

1. The abundance, which is the collected total count of each species
2. The density, estimated by dividing the total count of sandflies per night by the number of traps set per night
3. The relative frequency of each species estimated by dividing the collected number of a given species by the total count of all collected species multiplied by 100
4. The sex ratio, which is the ratio of males to females

The mean number of sandflies captured per trap per night per village was plotted on graphs to track seasonal fluctuations.

## 3. Results

### 3.1. Diversity and Abundance of Phlebotomine Species

In total, 4504 sandflies (2369 males (52.6%) and 2135 females (47.4%)) were collected during the study period from the two localities.

The following 13 species belonging to genera *Phlebotomus* and *Sergentomyia* were identified: *Phlebotomus* (*Phlebotomus*) *papatasi* Scopoli; *Ph.* (*Phl.*) *bergeroti* Parrot; *Ph.* (*Par.*) *alexandri* Sinton; Ph. (*Par.*) *sergenti* Parrot; *P.* (*Par.*) *chabaudi* Croset, Abonnec et Rioux; *Ph.* (*Par.*) *kazeruni* Theodor and Mesghali; *Ph.* (*Lar.*) *longicuspis* Nitzelescu; *Sergentomyia* (*Sergentomyia*) *schwetzi* Adler; *Se.* (*Ser.*) *minuta* Rodani; *Se.* (*Ser.*) *fallax* Parrot; *Se.* (*par.*) *africana* Newstead; *Se.* (*Gra.*) *dreyfussi* Parrot; and *Se.* (*Sin.*) *clydei* Sinton.

The abundance, the relative frequency, and the sex ratio were calculated for each species, and the results are given in Table 1.

The most abundant species overall in Tinzouline was *Ph. papatasi* (33.6%); *Ph. longicuspis* was the second most abundant species (25.7%) then *Ph. alexandri* (25.6%). These three species account for 84.9% of all sandflies collected in this survey.

*Phlebotomus papatasi* was the most abundant species in Mougni locality (44.7%), while *Ph. alexandri* was most abundant in Touna locality (41.8%).

The relative abundance of *Ph. longicuspis* was (19.8%) and (31.7%), respectively, in Touna and Mougni localities. *Ph. sergenti* was detected in the two localities with low relative abundance (less than 3%).

A total of 480 specimens belonging to *Sergentomyia* genus was found. *Se. fallax* was the most abundant *Sergentomyia* species (with relative abundance 4.6% among all sandflies).

In *Ph. papatasi*, *Ph. sergenti*, *Se. fallax*, and *Se. shwetzi* species, males clearly predominated. The ratio of males to females was, respectively, 1 : 0.6, 1 : 0.3, 1 : 0.5, and 1 : 0.6. This contrasts with *Ph. longicuspis*, *Ph. alexandri*, *Ph. bergeroti*, and *Se. dreyfussi* species, where females prevailed with, respectively, 1 : 1.2, 1 : 1.2, 1 : 6.9, and 1 : 2.9.

### 3.2. Sandfly Seasonal Fluctuations

**Figure 2:**
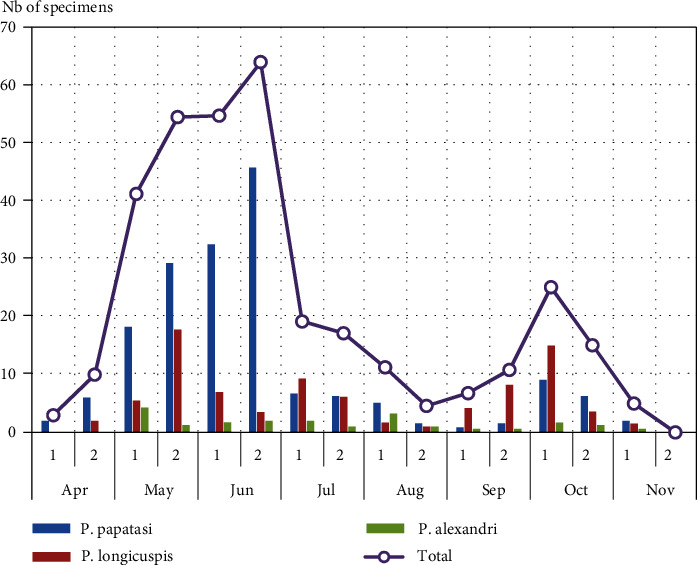
Bimonthly capture records for sandflies in Mougni locality.

**Figure 3:**
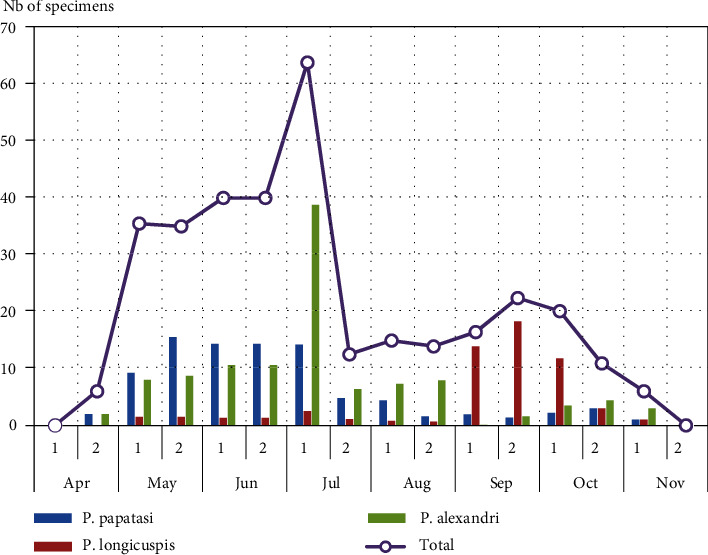
Bimonthly capture records for sandflies in Touna locality.

Figures 2 and 3 show the bimonthly capture records for total sandflies as well as for the 3 most abundant species, *Ph. papatasi*, *Ph. longicuspis*, and *Ph. alexandri*.

In both localities, the seasonal activity of sandflies extended from April to November. Abundance was highest during the months May, June, and July and lowest in August, September, and October. Total sandfly captures over the year showed two peaks, one in June-July and one, less important, in late-September-October. Seasonal abundance of adult *Ph. papatasi* in Mougni locality reflected a first peak in June, and then sandfly numbers decreased steadily in July-September as the climate became hotter, then increase slightly to mark a second peak clearly less important than the first in late-September-October. In Touna locality, *Ph. papatasi* showed a similar trend, but with only one peak in early July.

The bimonthly abundance of *Ph. longicuspis* and *Ph. alexandri* varied greatly between the two localities. *Ph. longicuspis* showed a bimodal peak pattern in Mougni locality: one in May and one in October, while it showed only one peak in September-October in Touna locality. *Ph. alexandri* was more abundant in Touna locality, showing one peak towards the beginning of the summer in June-July. In Mougni locality, it has no distinct seasonal pattern, since it was found in low numbers during the entire study period.

## 4. Discussion

Zagora province has long been known as an endemic CL focus, and the causative agent has been identified as *Leishmania major* [19]. This paper reports the results of the first entomology-based study conducted in this province in order to provide ecological data regarding the sandfly vector, *Ph. papatasi*, and help implement successful control measures.

In this study, thirteen sandfly species were identified from Tinzouline district out of the 24 species previously described in Morocco [20]. The identified species of the genus *Phlebotomus* belong to the three known subgenus, *Phlebotomus* (*Ph. papatasi* and *Ph. bergeroti)*, *Paraphlebotomus* (*Ph. sergenti*, Ph. *alexandri*, *Ph. chabaudi*, and *Ph. kazeruni*) and *Larroussius* (*Ph. longicuspis*). All of them have been already observed in previous studies in southern Morocco [13, 21–23].

The six species belonging to *Sergentomyia* genus (*Se. shwetzi*, *Se. minuta*, *Se. fallax*, *Se. africana, Se. dreifussi*, and *Se. clydei*) were weakly represented in sandfly fauna caught in this area. This finding is in line with observations made in other Moroccan CL endemic areas. These species are known to be less abundant in anthropic environments mainly inside and in the vicinity of human dwellings [13–17].

Except for *Ph. chabaudi* and *Ph. kazeruni* that were observed in Touna and not in Mougni, there was no difference in sandfly species diversity between the two villages. Three species encountered in the Tinzouline focus are involved in the transmission of the different *Leishmania* parasites. *Ph. papatasi* is considered the main vector of *L. major* in the south and southeast of the country, *Ph. longicuspis* is a known vector of *L. infantum* spread mainly in northern regions, and *Ph. sergenti*, the vector of CL caused by *L. tropica*, is reported mainly in the center of the country [5]. Two of these three species (*Ph. papatasi* and *Ph. longicuspis*) represented more than 59% of all collected sandflies, highlighting the risk for local disease transmission foci. *Phlebotomus sergenti* was scarce (2.7%). In a similar study conducted in a *L. tropica* focus in Tinghir province, bordering Zagora province, Faraj et al. [13] found that *Ph. papatasi* was the most abundant sandfly (45.7%), followed by *Ph. sergenti* (27.8%) then *Ph. longicuspis* (13.5%), while *Ph. alexandri* was the least-collected *Phlebotomus* species (1.1%).

*Phlebotomus papatasi* was found to be more abundant in the Mougni locality with 45% of the catch. It was less abundant in Touna (23%). This can be explained by the difference in altitude between the two localities; in fact, this species prefers to live in plain areas rather than in mountains [24]. In a study conducted in southwestern Morocco [25], authors reported negative association between altitude and abundance of *Ph. papatasi*. They indicated that this fly was predominant in plains (400–599 m), rare at the other altitudes, and absent from 1200 m a.s.l.

The adults of *Ph. papatasi* were active for eight months (April–November) with a bimodal evolution in Mougni and only one peak in Touna. Few studies have been done on the population dynamic of this species. The period of its activity was determined in Marrakech in the southwest where it was active throughout the year showing two peaks of density, the first in June and the second in November [11]. The same period of activity was postponed in Tinghir adjacent to Zagora, with two peaks in both June and August-September [13].

*Phlebotomus alexandri* was the most abundant species in Touna (42%). It was observed from April to November with one peak towards the beginning of the summer in July. This species prefers regions with a high percentage of relative humidity and warmer niches [26]. It is generally distributed in mountainous regions and sylvatic biotope [22].

*Phlebotomus longicuspis* flies were collected from April to November in the two localities showing a bimodal peak pattern in Mougni locality, one in May and one in October, and only one peak in September-October in Touna locality. The occurrence with considerable density and long activity period of this confirmed VL vector is a cause for concern and indicates the high potential risk of *L. infantum* transmission in the studied areas. In Tinghir province, this species showed only one peak in August [13], the same in Chichaoua province, southwest of Morocco; the density peak was in August-September [10]. According to Guernaoui et al. [25], *Ph. longicuspis* is more abundant between 600 and 799 m altitude while its density became much lower outside this range. These results are consistent with ours since this species was more abundant in the Mougni locality at an altitude of 775 meters.

## 5. Conclusion

This detailed study on the abundance and seasonal trends of *Ph. papatasi*, the vector of cutaneous leishmaniasis caused by *L. major* in Morocco, provides important baseline data for planning control interventions. According to the results of this study, control actions programmed twice a year at the time of density peaks would make it possible to control the transmission of the disease if these are correctly coordinated with case treatment and application of appropriate reservoir control. Raising public awareness of disease prevention measures is also important for the success of an epidemic control program. Entomological studies on the distribution and dynamics of vectors are important. They must be carried out regularly in different regions in order to provide decision-makers with up-to-date data.

## Figures and Tables

**Table 1:** Population, abundance, and sex ratio of sandfly species in the two stations studied.

	Touna Niaaraben					Ksar Mougni					Total		
	No. of collected sandflies			FR (%)	SR	No. of collected sandflies			FR (%)	SR	No. of collected sandflies (male/female)	FR (%)	SR
	Male	Female	Total			Male	Female	Total					
*Ph. papatasi*	274	243	517	22.7	1 : 0.9	666	331	997	44.7	1 : 0.5	1514	33.6	1 : 0.6
*Ph. longicuspis*	194	256	450	19.8	1 : 1.3	329	378	707	31.7	1 : 1.1	1157	25.7	1 : 1.2
*Ph. sergenti*	44	15	59	2.6	1 : 0.3	49	13	62	2.8	1 : 0.3	121	2.7	1 : 0.3
*Ph. alexandri*	458	493	951	41.8	1 : 1.1	72	129	201	9.0	1 : 1.8	1152	25.6	1 : 1.2
*Ph. chabaudi*	0	0	0	0	0	3	2	5	0.2	1 : 0.7	5	0.1	1 : 0.7
*Ph. bergeroti*	7	38	45	2.0	1 : 5.4	2	24	26	1.2	1 : 12.0	71	1.6	1 : 6.9
*Ph. kazeruni*	0	0	0	0	0	4	0	4	0.2	0	4	0.1	0
*Se. shwetzi*	40	22	62	2.7	1 : 0.6	16	9	25	1.1	1 : 0.6	87	1.9	1 : 0.6
*Se. minuta*	2	9	11	0.5	1 : 4.5	19	13	32	1.4	1 : 0.7	43	1.0	1 : 1.0
*Se. fallax*	84	29	113	5.0	1 : 0.3	59	36	95	4.3	1 : 0.6	208	4.6	1 : 0.5
*Se. africana*	2	8	10	0.4	1 : 4	9	7	16	0.7	1 : 0.8	26	0.6	1 : 1.4
*Se. dreyfussi*	4	19	23	1.0	1 : 4.8	10	22	32	1.4	1 : 2.2	55	1.2	1 : 2.9
*Se. clydei*	3	31	34	1.5	1 : 10.3	19	8	27	1.2	1 : 0.4	61	1.4	1:.1.8
Total	1112	1163	2275	100	1 : 1.04	1257	972	2229	100	1 : 0.8	4504	100	1 : 0.9

FR: relative frequency; SR: sex ratio.

## Data Availability

The data used in this study are included within the article.
